# A Study on the Effects of Rumen Acidity on Rumination Time and Yield, Composition, and Technological Properties of Milk from Early Lactating Holstein Cows

**DOI:** 10.3390/ani9020066

**Published:** 2019-02-21

**Authors:** Sudeb Saha, Luigi Gallo, Giovanni Bittante, Stefano Schiavon, Matteo Bergamaschi, Matteo Gianesella, Enrico Fiore

**Affiliations:** 1 Department of Agronomy, Food, Natural resources, Animals and Environment (DAFNAE), University of Padova, Viale dell’Università 16, 35020 Legnaro (PD), Italy; sudeb.saha@studenti.unipd.it (Su.S.); bittante@unipd.it (G.B.); stefano.schiavon@unipd.it (St.S.); matteo.bergamaschi@unipd.it (M.B.); 2 Department of Animal Medicine, Production and Health, University of Padova Viale dell’Università 16, 35020 Legnaro (PD), Italy; matteo.gianesella@unipd.it (M.G.); enrico.fiore@unipd.it (E.F.)

**Keywords:** dairy cows, rumen acidity, volatile fatty acids, rumination time, milk yield and composition, milk coagulation properties, cheese yield

## Abstract

**Simple Summary:**

The increase in milk yield achieved in recent decades by the dairy sector has been sustained by feeding dairy cows with more concentrates and less forage. This leads to increasing rumen acidity, a status widespread in high-producing dairy cows that may affect feed intake, impair ruminal digestion, and cause diarrhea, laminitis, inflammation, and liver abscesses. The effects of rumen acidity on milk yield and composition are controversial, while those on milk coagulation properties and cheese yield have not yet been explored. This study investigated whether the rumen acidity status affects rumination time, and the production, composition, coagulation properties and cheese yield of milk obtained by 100 early-lactating Holstein cows. The variation in rumen acidity was associated with changes in the cows’ rumen fluid composition and circadian pattern of rumination time. Moreover, daily milk yield linearly decreased as the rumen acidity increased. Conversely, the composition and technological properties of milk were unaffected, even when there were differences in rumen acidity, suggesting that variation in rumen acidity has little impact on cheese-making traits.

**Abstract:**

The use of high grain rations in dairy cows is related to an increase in rumen acidity. This study investigated whether the rumen acidity status affects rumination time (RT), and the production, composition, coagulation properties (MCPs) and cheese yield (CY) of milk. One hundred early-lactating Holstein cows with no clinical signs of disease and fed total mixed rations were used. Rumen fluid was collected once from each cow by rumenocentesis to determine pH and volatile fatty acid (VFA) content. The cows were classified according to the quartile of rumen acidity (QRA), a factor defined by multivariate analysis and associated with VFA and pH. Rumen fluid pH averaged 5.61 in the first quartile and 6.42 in the fourth, and total VFA content increased linearly with increasing rumen acidity. In addition, RT increased as rumen acidity increased, but only in the daily time interval from 08:00 to 12:00. Milk yield linearly decreased as rumen acidity increased, whereas QRA did not affect pH, fat or protein contents of milk. Furthermore, the MCPs, assessed by lactodynamograph, and CY were unaffected by QRA. It is suggested that differences in rumen acidity have little influence on the nutrient content, coagulation properties and CY of milk.

## 1. Introduction

The increase in milk yield achieved in recent decades by the dairy sector has been largely sustained by a concurrent increase in the use of concentrates in the rations for dairy cows [1]. This may lead to increasing rumen acidity, as an excess of grain induces rumen microorganisms to convert carbohydrates into volatile fatty acids (VFAs) at a rate exceeding rumen absorption, buffering and outflow capacity [1], with a concomitant reduction of the rumen fluid pH and possible rise in ruminal lactic acid concentration.

Periods of moderate rumen pH depression are defined as subacute ruminal acidosis (SARA) [2]. Field diagnosis of SARA is primarily based on the measurement of the ruminal pH [3], but diagnostic uncertainty arises from disagreements over the rumen pH threshold and the duration of periods of pH depression [1,2]. A drop in ruminal pH to non-physiological levels seems widespread [4,5] and is regarded with particular concern by the dairy industry [6]. Depressed rumen pH has been linked to lower feed intake [7] and milk yield [8] and an increased risk of liver abscesses and lameness [1]. Rumination time (RT) could also be associated with depressed rumen pH, as it is related to saliva production, which can help to buffer ruminal fluid [2].

The overall acidity status of the rumen content is expressed not only by the pH, but also by the amount and concentration of different VFAs in the rumen fluid, which in turn are related to the saliva production and rumination activity. Rumen acidity can alter the rumen microorganism composition, which affects the ruminal fermentation pattern and the acetate to propionate ratio, leading to alterations in milk components, particularly the milk fat content [9]. The association between ruminal pH, VFA concentration, and milk composition is controversial, as some authors found that rumen pH depression is related to a reduction in milk fat content [7], while others did not [5,10]. Rumen VFAs are the basis of the de novo synthesis of fatty acids in the udder [11], and changes in the milk fatty acids profile due to alterations in rumen biohydrogenation of polyunsaturated fatty acids, which occurs in the presence of low ruminal pH and may depress milk fat synthesis [12], could also affect milk coagulation properties. The nutrient content and technological properties of milk are critical issues for the dairy chain in areas where the majority of milk is processed into cheese, such as in Europe. However, to our knowledge, the effects of rumen acidity levels on the technological cheese-making properties of milk have not yet been explored.

Starting from the hypothesis that a variation in rumen pH and the correlated rumen fluid composition are associated with RT and the technological properties of milk, this study aimed to explore whether dairy cows with different single point ruminal acidity status would differ in the pattern of rumination, and in the production, composition, coagulation properties and cheese yield (CY) of milk.

## 2. Materials and Methods

### 2.1. Farms and Animals

The protocol of this study was compliant with the Italian legislation on animal care (DL n. 26, 4 March 2014). Collection of biological samples was performed by a skilled veterinarian under a sanitary inspection protocol of dairy herds aimed at monitoring rumen acidosis status on early lactating cows. In Italy, sanitary routine inspection, including collection of biological samples, does not require authorization, or an ID, or a protocol number. The study was conducted on 100 early-lactating Holstein cows (40 primiparous and 60 multiparous) kept in two commercial dairy herds in northern Italy. The farms, representative of the prevalent dairy system in the plains of the Veneto region, had an average herd size close to 150 lactating cows and an average milk yield close to 10,000 kg/cow/lactation. In both farms, cows were fed total mixed rations (TMRs), loosely housed in cubicle stalls, and milked twice a day, and their milk was destined for the production of typical hard cheeses. The composition of the TMRs and their nutritional contents, computed according to NRC 2001 [13], are given in Table 1. The cows were fed ad libitum through a mixer wagon in a single distribution at around 07:00 h.

Samples were collected once from 10 different cows in each of the 10 different recording sessions (6 in one herd and 4 in the other), so 100 cows were sampled during the study. At the beginning of each sampling session, the health status of the cows in the first 80 days in milk (DIM) was assessed on the basis of rectal temperature, heart rate, respiratory profile, appetite and faecal consistency. From these, 10 cows, primiparous and multiparous, with no obvious signs of clinical disease, were randomly selected for sampling.

### 2.2. Experimental Procedures

In each recording session, the cows were monitored for RT through external sensors over 5 consecutive days. Rumen fluid was collected once from each cow through rumenocentesis on the 3rd day of the recording session. On the same day as the rumenocentesis, the cows’ milk yield was recorded, a milk sample was collected and a body condition score (BCS), evaluated according to Edmonson et al. 1989 [14] on a scale ranging from 1, very thin, to 5, very fat, in 0.25 increments, was assigned by a skilled university technician having vast experience on scoring dairy cows for experimental purposes [15].

### 2.3. Rumination Time

The RT was recorded with RuminAct^TM^ (Milkline®, Podenzano, Italy), a microphone-based rumination monitoring system able to record the sounds of regurgitation and rumination. The system was used in previous research to measure the time spent ruminating during the day by dairy cows [16]. The RuminAct^TM^ microphone was fitted to the left side of the neck of each cow by a collar, and counted the minutes spent ruminating in 2-h intervals (min/120 min) from 00:00 to 24:00 h. Individual data were used to compute the circadian pattern of RT, expressed as the RT for each 2-h interval from 00:00 to 24:00 h (min/120 min, 60 records/cow) [16]. 

### 2.4. Rumen Fluid Sampling and Analysis

Rumen fluid (20 mL) was collected using a 50 mL syringe and a 13G 105-mm needle (Intralune PP, Vygon, France) inserted into the ventral sac of the rumen [4]. Rumen sampling procedures started around 5 hours after feed distribution, the recommended sampling time-point for ruminal fluid collection [3].

The pH of the rumen fluid was determined immediately after sampling, using a portable digital pH meter (Zetalab PC70; XS instruments, Padova, Italy). An aliquot of 8 mL of rumen fluid was immediately acidified with 2 mL of hydrogen chloride (HCl 0.6 M) and refrigerated at 4 °C until the samples arrived at the laboratory, where they were stored at −80 °C until analysis. The VFA and lactic acid contents were measured on the supernatant of the rumen fluid samples obtained by centrifugation (1300× *g* for 15 min) using an HPLC Perkin Elmer Series 10, mobile phase H_2_SO_4_ 0.0025 N, flux 0.6 mL/min, detector Waters 410, column Gecko 2000 at a working temperature of 60 °C [4].

### 2.5. Milk Sampling and Analysis

Milk samples (100 mL) were taken from each cow during the morning milking and stored in a refrigerator at −20 °C until analysis. Due to problems that occurred during the refrigeration of the samples, the composition and technological properties of the milk of 76 cows were determined. Milk composition (fat, protein, casein, lactose and total solids, respectively) was measured with a Milkoscan FT2 infrared analyser (Foss Electric A/S, Hillerød, Denmark). Milk pH was measured using a Crison Basic 25 electrode (Crison Instruments SA, Barcelona, Spain). Somatic cell count (SCC) was obtained with a Fossomatic Minor FC counter (Foss Electric A/S). Milk coagulation properties (MCPs) and CY were assessed in duplicate for each cow according to the 9-MilCA method [17] using 2 computerized lactodynamographs (Formagraph; Foss Electric A/S). The milk samples (9 mL) were placed in a Formagraph rack (8 samples per rack), heated to 35 °C for 15 min and mixed with 0.2 mL of rennet solution (Hansen Standard 215, 215 IMCU/ mL with 80 ± 5% chymosin and 20 ± 5% pepsin; Pacovis Amrein AG, Bern, Switzerland) diluted in distilled water (1.2 % wt/vol). The rack was moved to the lactodynamograph and observed for 30 min (120 curd firmness measures taken from each milk sample, 1 every 15 s) to measure traditional MCP traits (RCT, k_20_ and a_30_) and to obtain curd firming equation parameters (RCT_eq_, k_CF_ and CF_P_) according to Bittante 2011 [18]. The gelated milk samples were double-cut and heated for 30 min to 55 °C. The whey was drained from the curd, and analyzed for fat, protein, lactose and total solids content using FT2 (Foss Electric A/S, Hillerød, Denmark). The energy content of the milk and the whey was calculated as proposed by the NRC 2001 [13]. Three CY traits (CY_CURD_, CY_SOLIDS_ and CY_WATER_) and 4 milk nutrient recovery traits in the curd (REC) traits (REC_PROTEIN_, REC_FAT_, REC_SOLIDS_ and REC_ENERGY_) were determined from the weight and composition of the milk and the whey.

### 2.6. Editing Procedures and Statistical Analysis

A multivariate factor analysis was conducted to analyze the rumen fluid traits, including as variables the molar concentrations of acetate, iso-butyrate, normal-butyrate, propionate, isovalerate, normal-valerate, lactate, and the pH. The original variance of each trait was decomposed into its common and unique components, named as communality (BB′) and uniqueness (Ψ), using the SAS FACTOR procedure (SAS Institute, Inc., Cary, NC, USA), as detailed by Mele et al. 2016 [19]. The number of latent explanatory factors to be extracted was based on the Eigen values (>1), reliability in terms of relationships with the original variables, and the amount of explained variance. Factor reliability was improved through VARIMAX rotation. A variable was considered associated with a specific factor if the absolute value of its loading was ≥ 0.60. Two latent factors were extracted (Table 2): factor 1 was positively associated with acetate (load = 0.85, where load expresses the correlation between the latent factor and the measured trait), propionate (load = 0.75), iso-butyrate (load = 0.75), and lactate (load = 0.60), and negatively with pH (load = −0.78); factor 2 was positively related to normal-butyrate, iso-valerate and normal valerate. Factor 1 was associated with rumen acidity and used to compute a rumen acidity score for each cow, according to the following formula [19]:

X′ = y′ × (BB′ + **Ψ**)^−1^ × B (1)

where x′ is the row vector of the factor 1 scores, y′ is the row vector of standardized traits [(value − mean)/standard deviation)], and B represents the corresponding loading elements of the BB′ matrix of the theoretical factor variance model.

On the basis of the quartile of the rumen acidity score (QRA), the cows were classified in four groups having the same number of observations and ranging from the greatest to the lowest rumen acidity level moving from the 1st to the 4th QRA. The records were also classified for herd-test date (HTD, 10 classes, 10 cows/class), parity (PAR, primiparous and multiparous, 40 and 60 cows, respectively), and DIM (2 classes, DIM ≤ 35, 46 cows, and DIM > 35, 54 cows). Records relating to the technological properties of the milk were also classified for the position of the vat in the Formagraph racks (8 vats per rack, a total of 16 classes). After editing for missing values, the final data set for RT included 3096 records and 52 cows. Records of RT (min/120 min) were classified according to the day of observation (DAY, 5 classes from the day 1 to day 5) and for the 2-h daily time interval of collection within each DAY (HOURS, 12 intervals from 00:00 to 24:00 h).

Data were analyzed using a mixed model procedure (SAS 9.4), which included different effects according to the trait category being analyzed, namely: 

- for rumen fluid data (lactic acid and VFA content and proportion): the random effect of HTD and the fixed effects of PAR, DIM and QRA;

- for BCS, milk yield and milk composition data: the random effect of HTD and the fixed effects of PAR, DIM, and QRA; and

- for the technological properties of milk (MCPs and CY, for which 2 replicates per cow were available): the random effects of HTD and cow, and the fixed effects of PAR, DIM, vat, and QRA.

Polynomial contrasts (*p* < 0.05) were estimated between the least squares means of QRA in order to examine the response curve of the data with changing rumen acidity; the first-order comparisons measured linear relationships, whereas the second- and third-order comparisons measured quadratic and cubic relationships, respectively.

The circadian evolution of RT (min/120 min) was analyzed using the SAS MIXED procedure and a repeated measures model to allow for heterogeneous variances and correlations among different days. The model included the random effects of HTD and cow and the fixed effects of PAR, DIM, QRA, HOURS, DAY, and the QRA–HOURS and HOURS–DAY interactions. The analysis was carried out using compound symmetry as covariance structure, as it provided the least Akaike’s Information Criteria [20]. As the QRA–HOURS interaction was significant (*p* = 0.04), polynomial contrasts were estimated between the least squares means of the interaction in order to examine the response curve of RT within the HOUR with changing rumen acidity.

## 3. Results

### 3.1. Rumen Fluid Characteristics

As expected, rumen fluid pH was related to QRA and linearly decreased moving from the 4th to the 1st quartile (Table 3), characterized by a mean rumen fluid pH of 6.42 and 5.61, respectively. Nearly 32% and 14% of cows had a rumen fluid pH < 5.8 and < 5.6, respectively (data not in table). The total VFA content in the rumen fluid linearly increased moving from the 4th to the 1st quartile (*p* < 0.001), and was 40% greater in cows in the 1st than in those in the 4th QRA. In addition, the lactate content was influenced by QRA (*p* = 0.003), and reached the highest average value in cows of the 1st QRA, characterized by the highest rumen acidity. Acetate and propionate concentrations in the rumen fluid, which averaged 58% and 24% of total VFA, respectively, were significantly affected by QRA. Namely, the proportion of acetic acid linearly decreased and that of propionic acid linearly increased moving from the 4th to the 1st quartile (*p* < 0.001). As a consequence, the C2 to C3 ratio also decreased linearly as QRA decreased (*p* < 0.001), with average values close to 3 in the 4th and close to 2.2 in the 1st quartile, respectively.

### 3.2. Rumination Time

**Figure 1:**
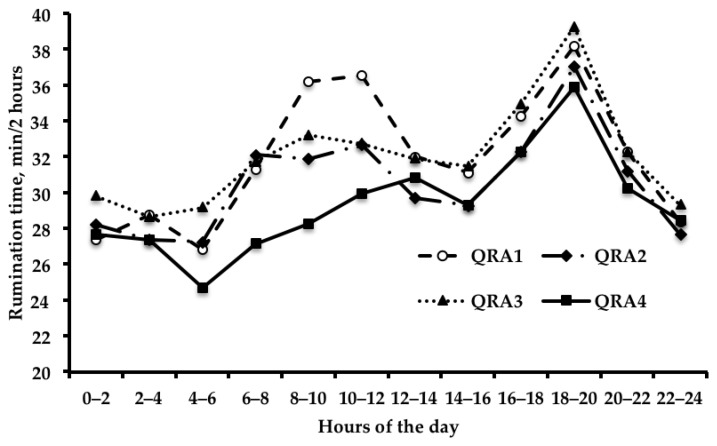
Effect of the quartile of rumen acidity score (QRA1, greatest rumen acidity, to QRA4, lowest rumen acidity) on the time spent in rumination (min/2 h) during the day by lactating Holstein cows (*p* value of contrast relative to linear effect of QRA: * < 0.05; SEM ranged from 1.81 to 1.85 min/2 h).

Rumination time exhibited a clear circadian variation (Figure 1), with two peaks: one at 10.00 to 12.00 h and one at 18.00 to 20.00 h. On average, cows spent nearly 375 min/day ruminating. QRA did not affect the average RT during the day (*p* > 0.05), even though it nominally increased as QRA decreased, and was nearly 8% greater in cows in QRA1 compared to those in QRA4 (data not shown in table). Conversely, we found a significant QRA–HOURS interaction (*p* = 0.04), that suggests an influence of rumen acidity on the circadian rumination pattern. Namely (Figure 1), RT linearly increased moving from the 4th to the 1st quartile during the daily time interval 08:00 to 12:00 (*p* < 0.05), but was similar across different QRA during the rest of the day.

### 3.3. Body Condition Score, Milk Yield and Milk Composition

**Figure 2:**
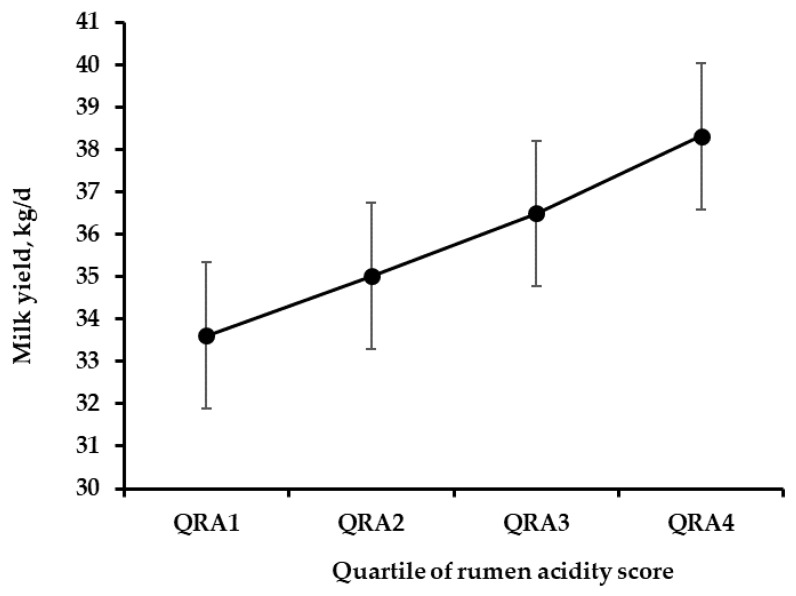
Effect of the quartile of rumen acidity score (QRA1, greatest rumen acidity, to QRA4, lowest rumen acidity) on milk yield (*p* of the linear component: < 0.03).

Milk yield averaged 35.8 kg/d and linearly decreased as rumen acidity increased (*p* < 0.03, Figure 2), so cows in the 1st quartile produced nearly 4.5 kg/d less milk than those in the 4th quartile. Conversely, QRA did not affect the cows’ BCS, which was on average close to 3 regardless of the level of rumen acidity (Table 4). The fat and protein content of milk approached 3.20 and 3.05%, respectively, and were also not influenced by QRA. Additionally, the somatic cell score appeared unaffected by QRA.

### 3.4. Milk Coagulation Properties and Cheese Yield Traits

On average, the rennet coagulation time (RCT) was close to 20.1 min. A curd firmness of 20 mm (k_20_) was attained after almost 6.0 min from gelation, and the curd firmness 30 min after rennet addition (a_30_) was 21.6 mm (Table 5). The mean values of the curd firming parameters were 19.2 min for the coagulation time calculated on the basis of all data points available (RCT_eq_); 40.1 mm for the asymptotic potential curd firmness theoretically achievable at infinite time in the absence of curd syneresis (CF_P_); and 7.6 %/min for the instant rate constant of curd firming (k_CF_). None of the traits relating to the coagulation properties of milk differed across different QRA.

On average, milk samples yielded nearly 14.4% of fresh cheese, comprising 36% solids and 64% water (Table 6). Milk protein, fat, solids and energy recoveries in the fresh cheese averaged 78, 64, 43 and 54%, respectively. QRA did not affect cheese yield or recovery traits. 

## 4. Discussion

### 4.1. Rumen Parameters and Rumination Time

Ruminal acidosis is a nutritional disorder of high-producing dairy cows, particularly in early lactation, when the energy density of diets is increased to meet their high nutritional requirements, and feeding excessively fermentable diets increases the risk of depressed rumen pH. The diagnosis of ruminal acidosis is mainly dependent on the monitoring of rumen pH, so rumenocentesis is usually performed in field trials to collect rumen fluid samples [12,21]. However, diagnosis is difficult in farm conditions as ruminal pH is subject to daily fluctuation and a single time-point measurement is not accurate enough to assess long lasting pH depression [3,7]. Given these difficulties, rather than referring to the possible and questionable SARA condition, in this study we combined the single point measures of the rumen fluid pH and VFA composition in a latent variable representing rumen acidity and we used this variable to investigate the relationships between the rumen acidity level and RT, composition and technological properties of milk.

Nearly 32% of cows had a rumen pH lower than 5.8, and in nearly 14% it was lower than 5.6. These values are consistent with data from field surveys where cows were sampled once for ruminal fluid [1,4,5]. The total VFA content increased linearly moving from the 4th to the 1st QRA, confirmation that the depression of rumen pH was mainly the consequence of an increased production of VFA in the rumen and reduced absorption of VFA [1]. Moreover, the increase in rumen acidity was associated with a linear decrease in the concentration of acetate and with a linear increase in propionate in the rumen fluid, the consequence of a variation in the cellulolytic and the amylolytic bacterial activity due to change in the ruminal acidity condition [9]. These variations may have a negative effect on the de novo synthesis of fatty acids (≤ 16 chain carbons) in the udder, because acetate is the primary precursor of fat synthetized by the mammary gland [11].

Plaizier et al. 2008 [1] stated that ruminal pH affects the growth of microbial populations and the physiological functions of the rumen, and it is also possible that greater rumen acidity would be related to different RT. In fact, differences in RT can affect saliva secretion, which seems to be greater during eating than during resting [22] and helps to stabilize ruminal pH by buffering the organic acids produced during the fermentation of carbohydrates [23]. There is growing interest in using ruminating behavior as a tool for early identification of health problems in dairy cows [24], although little is known about the relationship between rumen acidity and RT. The average daily RT and the circadian RT pattern observed in this study are similar to the figures and patterns reported by Schiavon et al. 2015 [16] for cows fed on similar rations and monitored using the same sensor. In the current research, we found that the average daily RT seems to be unaffected by differences in rumen acidity. However, the circadian pattern of RT was influenced by QRA, and the time spent ruminating increased linearly at increasing rumen fluid acidity after the morning feeding, when ruminal pH is expected to drop to its lowest value. DeVries et al. 2009 [6] observed that cows spent less time ruminating on the first day following an acidosis challenge, but more time on the second day and returned to pre-acidosis challenge levels thereafter. In a subclinical acidosis challenge trial, Khiaosa-ard et al. 2018 [25] observed that cows susceptible to SARA had longer ruminating and total chewing times compared with tolerant cows. Increased RT could, therefore, be interpreted as a sign of a cow’s resilience and a way to counterbalance the decrease in rumen acidity with increased saliva production.

### 4.2. BCS, Milk Yield and Composition

Subclinical acidosis has been associated with a lower and erratic feed intake [26], which may affect the cows’ BCS, although we did not find any relationship between QRA and BCS. Our results are in agreement with the findings of O’Grady et al. 2008 [27] and Bramley et al. 2013 [28], who also scored the cows’ condition at rumen fluid collection. It may be that a single time-point approach is not appropriate for traits that reflect long-term variation dynamic, such as BCS. Kleen et al. 2009 [29] also failed to find any significant relationship between the cows’ ruminal pH and their BCS measured once at rumenocentesis, but they observed that cows with SARA exhibited a greater fall in BCS over the calving period. 

The association between depressed rumen pH and milk production traits is rather controversial in the literature. In the current study, we found a strong communality among pH and some VFAs, but it is worth noting that pH explained only part of the total variance of the rumen acidity latent factor. In the current study, greater rumen acidity was related to a significant reduction in milk yield, and the magnitude of the response was similar to that reported by Stone 1999 [8], who found the milk yield of cows with SARA to be nearly 3 kg/d lower than that of normal cows. Conversely, in a field study carried out by O’Grady et al. 2008 [27], milk yield was found to be unaffected by rumen pH. Similarly, Kleen et al. 2013 [5] found no association between milk yield and the rumen pH in a survey of German herds, and Danscher et al. 2015 [7] did not find cows with induced SARA to have a lower milk yield.

Milk nutrient content appeared to be independent of rumen acidity in our study, despite the fact that a reduction in the acetate to propionate ratio in the rumen could result in the depressed synthesis of fat in the mammary gland [9]. Depressed ruminal pH has been frequently associated with a lower milk fat content [3,21]. Experimentally-induced subclinical acidosis reduced the milk fat percentage in some studies [7], but not in others [30]. Inconsistent results with respect to milk fat reduction after an acidosis challenge have been ascribed to the bouts of low rumen pH being too short to affect the milk fat content [30]. However, field studies have also frequently shown inconsistent relationships between rumen pH and milk fat content [5,27], and in a review of data from field studies, Kleen and Cannizzo 2012 [12] reported that milk fat depression was not prevalent in herds or cows with SARA.

### 4.3. Milk Coagulation Properties and Cheese Yield

Milk coagulation properties are commonly assessed using a lactodynamography to record three single-point measures (RCT, k_20,_ and a_30_) useful to the dairy industry [18]. Nearly 10% of the samples in our study failed to coagulate within 30 min of rennet addition, a figure comparable to the proportion of non-coagulating samples reported by Cecchinato et al. 2011 [31] in a large survey carried out on Holstein cows. The average RCT and k_20_ we observed were similar to those found by Malchiodi et al. 2014 [32] in purebred Holstein cows’ milk, whereas our a_30_ estimates were lower. Although widely used for assessing the technological traits of milk, traditional MCP parameters have certain limitations, such as the incidence of samples that do not coagulate within 30 min, precluding estimation of the RCT, k_20_ and a_30_, and the incidence of late-coagulating samples, precluding the estimation of k_20_ [18]. These limitations are partly overcome by a new procedure proposed by Bittante 2011 [18], in which the curd-firming process is modeled over time, so even samples with very late coagulation or very slow curd firming can be analysed. 

Neither the traditional MCP traits nor the curd-firming parameters estimated using the modeling equation were affected by QRA in our study, even though milk technological properties are influenced by several factors that may be related to ruminal conditions. It is well known that MCPs depend on milk composition, and that fat content is one of the main factors influencing the technological ability of milk [33]. Milk pH, too, may impact the technological properties of milk [34]. However, neither milk composition nor milk pH were affected by ruminal acidity in our study, and this can at least partly explain the lack of relationships found between MCPs and ruminal acidity. Moreover, Bittante et al. 2015 [35] and Stocco et al. 2017 [36] found some differences in the RCT and curd firming patterns between the milk produced by low-input herds and that produced by more intensive herds, which may also differ in the amounts of concentrates fed to the cows and consequently in rumen fluid conditions. The extent to which these findings may be related to rumen acidity is unknown. The relationships between rumen fluid acidity and the technological characteristics of milk have not been explored yet, and further research in this area is needed.

The efficiency of the cheese-making process relies heavily on CY as well as on the recovery of individual milk constituents in the curd and their loss in the whey. Cheese yield traits are influenced by several dairy cow-related factors, such as breed, milk composition and MCPs, and farming conditions [37], but there has been little investigation so far of the effects of common health disorders on CY, particularly at individual cow level. This is relevant for conditions that seem to be widespread in commercial herds, such as those related to an increase in rumen fluid acidity. The average fresh CY (14.3%) we found is comparable to the 14.1% CY predicted by Cecchinato et al. 2015 [38] for Holstein Friesian cows, and both studies had similar recoveries of protein in the curd, although we found a greater proportion of water to solids in the curd, resulting in lower recoveries of fat and solids. Neither cheese yield nor nutrient and energy recoveries were influenced by rumen fluid acidity, even though the cows in the bottom QRA, characterized by an average rumen fluid pH close to 5.6, produced milk with a nominally greater CY and nutrient recovery in cheese than cows in the other QRA.

## 5. Conclusions

The results of this study confirm that the level of rumen acidity in early-lactating Holstein cows with no obvious signs of clinical disease may be quite variable, at least in intensively-managed commercial dairy herds. The decrease in rumen pH has been associated with a linear increase in the content of all VFAs in the rumen fluid and with changes in the relative proportions of acetate and propionate, so a latent variable was extracted to represent the overall rumen acidity condition. Cows with different ruminal acidity spent a similar amount of time ruminating during the day, but their RT differed after the morning feeding. The time spent ruminating increased during the 08:00 to 12:00 h interval as rumen acidity increased. However, the usefulness of monitoring RT as a tool for identifying cows with acid rumen conditions needs to be revaluated with a larger number of cows. Even when there were differences in rumen acidity, the composition, coagulation, curd firming patterns and cheese yield of milk were unaffected, excluding a possible detrimental relationship between acid rumen fluid conditions and the properties of milk destined for cheese production. As the current study was carried out in field conditions, the single time-point rumenocentesis used to collect rumen fluid samples was unfit to measure the amount of time spent daily under the critical pH value, thus preventing the possibility of the reliable diagnosing of an eventual state of subacute ruminal acidosis. Therefore, further studies are needed to investigate more thoroughly the relationships between rumen pH depression and the technological properties of milk.

## Figures and Tables

**Table 1:** Characteristics of the rations used in the two farms.

Items ^1^	Farm A	Farm B
Feed ingredients, kg DM/d:		
Corn silage	5.95	4.76
Ear corn silage	3.90	4.23
Barley silage	-	1.65
Grass silage	1.81	1.18
Meadow hay	2.30	0.88
Alfalfa hay	-	0.57
Wheat straw		0.44
Corn meal	0.87	-
Soybean meal (Solv. Extr.)	2.46	2.20
Sunflower meal (Solv. Extr.)	1.35	0.90
Commercial feed mixture	0.87	2.22
Propylene glycole	0.40	-
Linseed seed	0.35	-
Flaked soybean seeds	0.35	-
NaCl	0.05	0.05
NaHCO_3_	0.05	0.05
Hydrogenated soybean oil	0.05	-
Total dry matter intake, kg DM/d	20.76	19.18
Chemical constituents, g/kg DM:		
Crude protein	169	168
NDF	372	394
ADF	218	234
Starch	230	236
Ether extract	37	28
Ash	34	39
Net energy for milk, MJ/kg DM	6.9	6.3

^1^ DM = dry matter; NDF = neutral detergent fibre; ADF = acid detergent fibre.

**Table 2:** Rotated latent explanatory factor pattern and the proposed factor names.

Items	Factor 1 “Rumen Acidity”	Factor 2 “Protein Degradation”	Communality	Eigen Value
Acetate	0.85	0.15	0.75	1.19
Propionate	0.75	0.25	0.62	0.62
Iso-butyrate	0.73	0.32	0.63	0.85
Lactate	0.60	0.01	0.36	0.22
Normal-butyrate	0.49	0.69	0.71	0.49
Iso-valerate	0.36	0.71	0.63	0.29
Normal-valerate	−0.04	0.93	0.86	0.25
pH	−0.78	−0.33	0.72	4.10
Cumulative variance	0.60	0.40		

**Table 3:** Effect of the quartile of rumen acidity score (QRA1, greatest rumen acidity, to QRA4, lowest rumen acidity) on pH, lactate and volatile fatty acid (VFA) content and proportion on the rumen fluid of Holstein cows.

Items	Quartile of Rumen Acidity Score				SEM	*QRA*, *p* Value	Contrasts, *p* Value		
	QRA1	QRA2	QRA3	QRA4			Linear	Quadratic	Cubic
pH	5.61	5.82	6.04	6.42	0.05	<0.0001	<0.0001	0.17	0.92
Lactate, mmol/L	0.974	0.201	0.178	0.003	0.221	0.01	0.003	0.17	0.35
VFA, total, mmol/L:	98.54	86.57	77.01	62.74	2.09	<0.0001	<0.0001	0.49	0.31
Acetic acid	54.48	48.28	44.90	39.06	0.85	<0.0001	<0.0001	0.82	0.13
Propionic acid	25.84	21.56	18.28	13.13	1.07	<0.0001	<0.0001	0.65	0.49
Iso-butyric acid	0.77	0.63	0.49	0.40	0.03	<0.0001	<0.0001	0.34	0.71
N-butyric acid	13.02	12.06	9.92	7.65	0.75	<0.0001	<0.0001	0.17	0.60
Iso-valeric acid	1.88	1.58	1.36	1.13	0.15	<0.0001	<0.0001	0.74	0.84
N-valeric acid	2.55	2.43	1.86	1.70	0.35	0.002	<0.0001	0.94	0.29
C2:C3 ratio	2.23	2.32	2.60	3.01	0.13	<0.0001	<0.0001	0.16	0.92
VFA, mmol/100 mol:									
Acetic acid	55.79	56.05	58.73	61.37	1.2	<0.0001	<0.0001	0.14	0.47
Propionic acid	26.00	24.95	23.55	20.94	0.86	0.0004	<0.0001	0.33	0.81
Iso-butyric acid	0.78	0.73	0.63	0.66	0.04	0.02	0.005	0.28	0.28
N-butyric acid	13.05	13.87	13.01	12.04	0.72	0.12	0.51	0.66	0.12
Iso-valeric acid	1.88	1.81	1.79	1.79	0.17	0.95	0.63	0.78	0.99
N-valeric acid	2.56	2.74	2.43	2.83	0.36	0.27	0.52	0.49	0.08

**Table 4:** Effect of the quartile of rumen acidity score (QRA1, greatest rumen acidity, to QRA4, lowest rumen acidity) on the body condition score (BCS) and milk composition of Holstein cows.

Items	Quartile of Rumen Acidity Score				SEM	QRA, *p* Value
	QRA1	QRA2	QRA3	QRA4		
BCS	2.92	2.97	2.97	2.93	0.07	0.91
Milk pH	6.54	6.55	6.49	6.53	0.02	0.18
SCS ^1^	3.83	4.21	3.18	3.48	0.59	0.41
Fat, %	3.26	3.05	3.10	3.31	0.16	0.62
Protein, %	3.05	3.01	3.17	2.96	0.07	0.14
Lactose, %	5.03	5.03	5.08	5.05	0.05	0.75
Total solids, %	11.96	11.74	12.01	11.95	0.16	0.66
Fat: Protein ratio	1.08	1.01	0.99	1.13	0.06	0.25

^1^ SCS = log_2_ (SCC/100,000) + 3.

**Table 5:** Effect of the quartile of rumen acidity score (QRA1, greatest rumen acidity, to QRA4, lowest rumen acidity) on single point milk coagulation properties (MCPs) and the curd firming equation parameters of Holstein cows.

Items	Quartile of Rumen Acidity Score				SEM	*QRA,* *p* Value
	QRA1	QRA2	QRA3	QRA4		
Single point MCP ^1^:						
RCT, min	19.12	20.39	18.39	21.10	1.31	0.39
k_20_, min	6.33	5.53	4.76	5.41	0.80	0.50
a_30_, mm	24.72	22.30	29.34	21.15	4.01	0.35
Curd firming parameters ^2^:						
RCT_eq_, min	18.60	20.54	18.58	21.25	1.14	0.25
CF_p_, mm	38.85	35.56	38.26	42.52	4.48	0.68
k_CF_, %/min	12.33	12.13	14.15	10.70	0.95	0.09

^1^ Single point MCP: RCT = rennet coagulation time; k_20_ = time to a curd firmness of 20 mm; a_30_ = curd firmness after 30 min from rennet addition. ^2^ Curd firming parameters: RCT_eq_ = rennet coagulation time estimated using the equation; CF_P_ = asymptotic potential curd firmness; k_CF_ = curd firming instant rate constant.

**Table 6:** Effect of the quartile of rumen acidity score (QRA1, greatest rumen acidity, to QRA4, lowest rumen acidity) on cheese yield (CY) and milk nutrient and energy recovery in curd (REC).

Items	Quartile of Rumen Acidity Score				SEM	*QRA,* *p* Value
	QRA1	QRA2	QRA3	QRA4		
CY ^1^, %:						
CY_CURD_	14.70	13.98	14.59	14.27	0.43	0.56
CY_SOLIDS_	5.09	5.07	5.16	4.78	0.18	0.44
CY_WATER_	9.49	8.96	9.45	9.52	0.36	0.58
REC ^2^, %:						
REC_PROTEIN_	78.79	78.14	77.83	78.59	0.55	0.99
REC_FAT_	66.68	63.86	61.68	60.17	4.08	0.47
REC_SOLIDS_	43.46	42.92	41.60	40.83	1.02	0.68
REC_ENERGY_	54.57	53.84	54.22	52.48	1.61	0.80

^1^ CY_CURD_ = fresh cheese yield; CY_SOLIDS_ = total solids cheese yield; CY_WATER_ = water retained in the curd. ^2^ REC_PROTEIN_ = milk protein retained in the curd; REC_FAT_ = milk fat retained in the curd; REC_SOLIDS_ = total milk solids retained in the curd; REC_ENERGY_ = milk energy retained in the curd.
